# Coronary Intervention Outcomes in Patients with Liver Cirrhosis

**DOI:** 10.1007/s11886-024-02163-x

**Published:** 2025-01-04

**Authors:** Song Peng Ang, Jia Ee Chia, Jose Iglesias, Muhammed Haris Usman, Chayakrit Krittanawong

**Affiliations:** 1 Department of Medicine, Rutgers Health/Community Medical Center, Toms River, NJ USA; 2 Department of Medicine, Texas Tech University Health Science Center, El Paso, TX USA; 3 Department of Medicine, Hackensack Meridian School of Medicine, Nutley, NJ USA; 4 Department of Cardiology, Newark Beth Israel Medical Center, Newark, NJ USA; 5 Division of Cardiology, NYU Grossman School of Medicine, New York, NY USA

**Keywords:** Coronary intervention, Outcomes, Cirrhosis, Chronic liver disease, Bleeding, Acute kidney injury

## Abstract

**Purpose of Review:**

This review assesses the outcomes of coronary interventions in patients with liver cirrhosis and coronary artery disease (CAD), focusing on the clinical challenges posed by cirrhosis-related hemodynamic and coagulopathic changes. It highlights essential considerations for managing these patients, who have an increased risk of adverse events during coronary procedures.

**Recent Findings:**

Recent studies have shown that patients with liver cirrhosis undergoing PCI experience significantly higher mortality rates compared to non-cirrhotic patients, particularly in the context of STEMI and NSTEMI. Coagulopathy and thrombocytopenia increase the risk of bleeding and vascular complications during interventions. Radial access has been suggested as a safer alternative to femoral access in these patients due to reduced bleeding complications. Additionally, contrast-induced nephropathy (CIN) is a prevalent risk, with cirrhotic patients demonstrating higher rates of acute kidney injury post-PCI. Preventive strategies such as minimizing contrast exposure and utilizing intravascular ultrasound (IVUS) are recommended.

**Summary:**

Managing CAD in cirrhotic patients requires careful consideration of their unique pathophysiological state. Higher in-hospital mortality, bleeding risks, and vascular complications necessitate tailored procedural strategies, such as radial access and contrast minimization. The balance between thrombotic and bleeding risks is critical in decision-making, with IVUS and hydration strategies being promising approaches. Further research is required to optimize treatment protocols and improve long-term outcomes for this high-risk population.

## Background

Coronary artery disease (CAD) remains a predominant cause of death globally, with an estimated 17.9 million deaths each year attributed to cardiovascular diseases, primarily heart attacks and strokes [1]. Advances in medical therapy and revascularization techniques, such as percutaneous coronary intervention (PCI) and coronary artery bypass grafting (CABG), have significantly improved outcomes for many patients [2, 3]. However, managing CAD in patients with comorbid conditions like liver cirrhosis poses unique challenges due to their altered physiological state.

Liver cirrhosis represents the final common pathway of chronic liver injury from various etiologies, including chronic viral hepatitis, alcohol abuse, and non-alcoholic fatty liver disease (NAFLD). It is characterized by fibrosis and the formation of regenerative nodules, leading to portal hypertension and progressive liver dysfunction. Globally, cirrhosis accounts for over 1 million deaths annually and is the 11th leading cause of death [4, 5]. As patients with liver cirrhosis live longer due to advancements in medical care, the incidence of CAD in this population is expected to rise [6]. 

## Rationale

Patients with liver cirrhosis frequently present with coexisting CAD, with reported prevalence ranging from 2 to 37% [7–9]. The management of CAD in this population is particularly challenging due to the complex interactions between hepatic dysfunction and cardiovascular health. Liver transplantation, the only definitive treatment for decompensated cirrhosis, is associated with increased perioperative morbidity and mortality when CAD is present [10, 11]. Notably, approximately 40% of deaths within 30 days following liver transplantation are attributed to cardiac causes [12]. Consequently, current guidelines recommend comprehensive cardiovascular assessment, including CAD screening, for all cirrhotic patients being evaluated for liver transplantation [11, 13, 14]. This evaluation typically includes non-invasive stress testing, echocardiography, and, in select cases, coronary angiography to detect underlying cardiovascular disease that could complicate surgical outcomes. For patients with significant CAD, coronary revascularization may be considered to optimize cardiac function before transplantation [15]. However, cirrhotic patients are at heightened risk of complications during coronary interventions, including bleeding, thrombocytopenia, and hemodynamic instability. This review will examine the pathophysiological connections between cirrhosis and CAD, evaluate the outcomes of coronary interventions in this patient group, and summarize current recommendations for risk stratification and management of CAD in individuals with liver cirrhosis.

## Pathophysiology Linking Liver Cirrhosis and CAD

Liver cirrhosis induces systemic hemodynamic changes that significantly impact cardiovascular health. The hallmark of cirrhosis-related circulatory dysfunction is a hyperdynamic circulatory state characterized by increased cardiac output and decreased systemic vascular resistance [16]. This state results from vasodilatory substances, such as nitric oxide, being overproduced due to portal hypertension and endotoxemia.

Endothelial dysfunction is a critical link between liver cirrhosis and atherosclerosis [17, 18]. Metabolic dysfunction-associated steatotic liver disease (MASLD), previously known as non-alcoholic fatty liver disease, are recognized precursors to cirrhosis and are significant contributors to cardiovascular disease (CVD) risk. MASLD, characterized by ectopic fat accumulation in the liver, disrupts normal metabolic functions, leading to insulin resistance and systemic inflammation, both of which exacerbate atherosclerosis. As liver steatosis progresses to steatohepatitis, chronic inflammation and oxidative stress further damage endothelial cells, promoting vascular injury and accelerating atherogenesis [19]. Metabolic disturbances such as dyslipidemia, insulin resistance, and increased levels of pro-inflammatory cytokines are common in MASLD, compounding the risk of developing CAD [20, 21]. Research suggests that hepatic diacylglycerol accumulation, a hallmark of MASLD, is closely linked to insulin resistance and impaired endothelial function, ultimately contributing to increased CVD risk [22]. Additionally, oxidative stress and impaired nitric oxide bioavailability play critical roles in endothelial dysfunction, which hastens the development of atherosclerosis in these patients [19]. 

Cirrhotic cardiomyopathy, a condition characterized by impaired cardiac contractility and electrophysiological abnormalities, further complicates the scenario [23]. Patients may have a blunted cardiac response to stress, which can impact perioperative cardiac function during coronary interventions [24, 25]. 

## Short-term Outcomes of PCI

### Mortality

Studies have shown that patients with cirrhosis undergoing PCI have notably higher mortality rates compared to non-cirrhotic patients, with most data derived from the Healthcare Cost and Utilization Project (HCUP) national databases [26–28]. Alqahtani and colleagues investigated the outcomes of PCI among patients with and without cirrhosis using the National Inpatient Sample database from 2003 to 2016 [26]. They concluded that the use of PCI among liver cirrhosis has significantly increased over the years compared to that without cirrhosis (p-trend < 0.01). Their findings indicated a significant increase in the use of PCI over time in patients with liver cirrhosis compared to non-cirrhotic patients (p-trend < 0.01). In their unadjusted analyses, patients with liver cirrhosis experienced higher in-hospital mortality compared to non-cirrhotic patients. After propensity-score matching, patients with cirrhosis continued to have higher in-hospital mortality, specifically among those presenting with STEMI (19.1% vs. 11.5%), NSTEMI (8.7% vs. 5.6%), and stable ischemic heart disease (7.7% vs. 4.3%). Istanbuly and colleagues similarly utilized the NIS database to objectively assess the in-hospital outcomes of patients with and without cirrhosis undergoing PCI [28]. After multivariate adjustment, they reported that patients with cirrhosis had 43% higher odds of in-hospital mortality than those without cirrhosis, with the results being statistically significant. Further stratification based on cirrhosis severity revealed that patients with decompensated cirrhosis, characterized by hepatic encephalopathy, portal hypertension, hepatorenal syndrome, thrombocytopenia, or coagulopathy, had significantly worse in-hospital survival than those without these features (OR, 1.34, 95% CI 1.28–1.41, *p* < 0.001). Lu et al. utilized the National Readmission Database to examine approximately 7,000 patients with liver cirrhosis who underwent PCI [27]. Their results aligned with prior studies, demonstrating an elevated risk of in-hospital mortality among cirrhotic patients. In addition, they found that patients with cirrhosis had a significantly higher 90-day mortality compared to non-cirrhotic patients (10.3% vs. 2.5%).

### Bleeding

Cirrhotic patients undergoing PCI present distinct challenges, primarily due to the complex interplay of liver disease and procedural risks. One of the concerns is the increased risk of bleeding, driven by a combination of thrombocytopenia, impaired hemostasis, impaired platelet function and reduced levels of clotting factors [29–31]. Consequently, the presence of liver cirrhosis with portal hypertension is recognized as a major criterion for high bleeding risk in patients undergoing PCI. In particular, these patients are at least considered as Bleeding Academic Research Consortium (BARC) type 3 or 5, which corresponds to a bleeding risk of at least 4% at 1 year [32]. 

Supporting data highlight this elevated bleeding risk. In a nationwide analysis in the United States, Singh and colleagues investigated approximately 12,000 individuals with liver cirrhosis undergoing PCI and found that the most common complications were bleeding (6.6%) and need for blood transfusion (11.3%) [33]. Furthermore, Patel et al. analyzed over 4 million patients from the NIS registry and demonstrated that liver disease is an independent predictor of in-hospital gastrointestinal bleeding among those undergoing PCI (OR, 2.59 95% CI, 2.22–3.02, *p* < 0.001) [34]. In a multicenter registry-based study, Krill and colleagues compared cirrhotic patients with CAD who underwent PCI to those who were managed medically. The study found that cirrhotic patients who underwent PCI had a significantly higher 30-day risk of gastrointestinal bleeding compared to those in the medical management arm. This elevated bleeding risk became even more pronounced during the two-year follow-up period [35]. Therefore, heightened caution is warranted in cirrhotic patients, and the increased bleeding risk must always be balanced against an equally important assessment of thrombotic risk.

### Vascular Complications

The rates of vascular complications varied considerably, likely due to difference in patient population, case definitions and coding practice. Singh studied the trend of vascular complications and reported an overall rate of 1.48%, with annual rate ranging from 0.7 to 2.8% from 2005 to 2012 among cirrhotic patients who received PCI [33]. 

#### Vascular Access

When considering vascular access in this population, the selection of an appropriate access site is essential. In line with the recommendation for the preferential access site for the general population, radial access should be the default strategy in PCI for patients with cirrhosis, whenever feasible, to minimize complications. Femoral access has been shown to carry a higher risk of significant bleeding, particularly in patients with coagulopathies. For cirrhotic patients, this risk is amplified by their underlying hematologic derangements as aforementioned. In contrast, radial access presents a safer alternative, with multiple studies demonstrating its ability to reduce access site complications, including major bleeding events.

Data from large studies, such as the RAD-MATRIX trial, reinforce this preference [36]. Radial access in PCI was associated with significantly lower rates of major bleeding compared to femoral access (1.6% vs. 2.3%, respectively). Moreover, the benefit of radial access is even more pronounced in high-volume centers with substantial operator experience, highlighting the importance of procedural expertise in optimizing patient outcomes. Additionally, radial access has been linked to lower rates of major adverse cardiovascular events (MACE), further emphasizing its suitability in this high-risk population.

In relation to patients with cirrhosis, Feng and colleagues studied 300 patients with end-stage liver disease with transradial or transfemoral access for PCI [37]. The study demonstrated that trans-radial access was associated with significantly lower vascular complications, such as the absence of pseudoaneurysms, compared to trans-femoral access, despite higher baseline bleeding risks in the radial group. While the overall rates of major bleeding did not differ significantly, the reduction in vascular complications suggests that trans-radial access may offer a safer alternative for cirrhosis patients, who share similar bleeding risk profiles. Huded and colleagues compared the use of transradial access in liver transplant candidates to that of non-liver transplant candidates, with primary endpoint being radial approach failure [38]. In their analysis of 1,071 patients, they found no significant difference in primary endpoint and in the incidence of adverse events including hematoma and CIN between the two groups of patients. These findings support the consideration of trans-radial access as a preferred approach in cirrhosis patients undergoing PCI.

### Acute Kidney Injury (AKI)

Contrast-Induced Nephropathy (CIN) is a form of acute kidney injury (AKI) that occurs following the direct administration of iodinated contrast media, especially during arterial procedures such as PCI. The kidneys, which are the primary organs responsible for excreting contrast media, receive approximately 25% of the body’s arterial blood supply, making them highly susceptible to contrast-induced damage. CIN is characterized by a significant increase in serum creatinine, typically a 25% rise from baseline or an absolute increase of 0.5 mg/dL within 48 to 72 h after contrast administration. The mechanisms include direct nephrotoxicity and renal medullary ischemia due to altered renal hemodynamics. In the latter scenario, contrast agents induce vasoconstriction, especially in the renal microvasculature, primarily through the release of vasoconstrictors such as endothelin and the reduction in vasodilators including nitric oxide and prostaglandins. This vasoconstriction disproportionately affects the outer medulla, which is inherently prone to low oxygenation. The resulting ischemia impairs the ability of the medulla to regulate sodium and water balance, leading to a cascade of damage, resulting in acute tubular necrosis [39]. 

#### Epidemiology of CIN

Contrary to the CIN related to intravenous iodinated contrast, the risk of CIN following PCI is substantially higher. The risk of CIN in the context of the former was estimated to be close to 0% in individuals with eGFR greater than or equal to 45 and 0–17% for eGFR below 30. In contrast, the risk of CIN was estimated to be 13% according to a meta-analysis of 12 studies investigating the prevalence of CIN in STEMI patients undergoing PCI [40]. Several studies investigated the incidence of AKI following PCI in patients with liver cirrhosis. The risk of AKI following PCI in cirrhosis was reportedly to be around 22% in a study with approximately 4% of them requiring dialysis [27]. Bhandari and colleagues retrospectively analyzed 544 patients with end-stage liver disease who underwent coronary angiogram [41]. They reported an incidence of contrast-induced AKI of 23%. Baseline eGFR was found to be a significant predictor of development of contrast-induced AKI.

#### Risk Factors of CIN in Cirrhosis

Cirrhotic patients are at higher risk of developing CIN due to several factors. Hypovolemia is frequently observed in patients with decompensated cirrhosis, responsible for around 30-40% of acute kidney injury. Individuals with cirrhosis are particularly susceptible to volume depletion and prerenal azotemia due to several factors ranging from the use of diuretics in ascitic patients, diarrhea from laxatives used to prevent hepatic encephalopathy and reduced oral intake. Additionally, conditions intrinsic to cirrhosis, such as hypoalbuminemia and reduced effective arterial blood volume, increase the risk of prerenal azotemia [42, 43]. In the context of cardiac catheterization, the use of contrast further compounded the risk of contrast-induced nephropathy in these patients [44]. 

#### Preventive Strategies for CIN

Currently, a wide range of preventive strategies for CIN in patients undergoing coronary angiography have been explored. Periprocedural hydration and contrast minimization remained the principal strategies to lower the risk of CIN. Given the scarce data of CIN on the cirrhotic patients, insights from populations at similarly high risk, such as those with advanced chronic kidney disease (CKD) undergoing PCI, may be extrapolated.

#### Use of Ultra-low Contrast Volume

Data on the use of ultra-low contrast volume during angiography or PCI remained inconclusive. In the CONSaVE-AKI (Ultra-low contrast PCI vs. conventional PCI in patients of ACS with increased risk of CI-AKI) trial, there was significantly lower incidence of CIN among patients with ultra-low contrast PCI compared to those with conventional PCI (*p* = 0.012) [45]. In contrast, other real world studies have shown no significant differences in eGFR or serum creatinine levels before and after the procedure, with similarly high procedural success rates [46–48]. 

#### Use of Intravascular Ultrasound (IVUS)

The use of IVUS should be encouraged in these patients, as it has been associated with reduced contrast volume [49]. Data supporting IVUS come largely from the MOZART trial, which randomized 83 patients undergoing PCI to either angiography-guided or IVUS-guided procedures [49]. The IVUS-guided group demonstrated a significant reduction in total contrast volume compared to the angiography-guided group.

#### Hydration Strategies

While hydration remained a useful tool to prevent CIN in general population, volume expansion in patients in cirrhosis to prevent renal dysfunction remained controversial. The recent ATTIRE trial (A Randomized Trial of Albumin Infusions in Hospitalized Patients with Cirrhosis; ISRCT number, N14174793) which randomized hospitalized patients with decompensated cirrhosis to prophylactic albumin infusion and standard of care showed that there was no significant difference in the incidence of kidney dysfunction between both groups of patients [50]. Notably, the intervention group had a higher risk of pulmonary edema compared to the control group. These findings suggest that close assessment of volume status is crucial, and the use of albumin as a volume expander should be judicious, especially given the increased risk of pulmonary edema in patients with acute coronary syndrome.

### Long-term Outcomes of PCI

Data on the long-term outcomes following PCI in patients with liver cirrhosis is scarce. Lu et al. identified 42 cirrhotic patients who underwent PCI and compared them with 29 patients who were managed medically [51]. Both cohorts had angiographically significant CAD. In terms of severity of cirrhosis, there was no significant difference in terms of MELD, MELD-Na score and Child-Pugh class between the two groups. Over a follow-up period of one year, there was no significant difference in terms of MACE, defined as a composite of all-cause mortality, repeat revascularization and myocardial infarction (50% in PCI cohort vs. 40% in medical cohort, *p* = 0.38). Nevertheless, when evaluating each endpoint individually, those with PCI had a statistically higher risk of 1-year mortality compared to those who were managed medically (42% vs. 14% respectively), with otherwise no significant difference in the risk of myocardial infarction and repeat revascularization.

### Risk Prediction Score in PCI

Various risk prediction tools have been employed to forecast both short- and long-term outcomes of PCI in patients with liver cirrhosis. Studies have shown that the model for end-stage liver disease (MELD) score was an independent predictor of long-term mortality and cardiovascular mortality in this population [52–54]. Additionally, its derivative, the MELD-Albumin score, was also predictive of CIN following PCI [52]. However, much of the current evidence is based on retrospective studies, highlighting the need for prospective validation to confirm the utility and accuracy of these scores in clinical practice.

## CABG

PCI remains the preferred revascularization strategy for many patients with liver cirrhosis due to its minimally invasive nature and lower immediate surgical risks. An alternative mode of coronary revascularization is CABG, which have shown superiority in patients with left main coronary artery disease or multivessel disease in terms of long-term mortality and reduction in MACE compared to PCI in the general population. However, it remained unclear whether the benefit can be extrapolated to patients with liver cirrhosis of varying severity. CABG introduces distinct challenges and risks in this population that must be carefully weighed. This section will discuss the outcomes including postoperative complications of CABG in patients with cirrhosis.

### Outcomes of CABG

Shaheen and colleagues studied approximately 400,000 patients undergoing CABG, of which 711 patients had underlying cirrhosis [55]. Compared to non-cirrhotic patients, patients with liver cirrhosis had 6-fold higher risk of in-hospital mortality, after adjusting for confounders including patients’ demographics, comorbidities and use of cardiopulmonary bypass. Similarly, Gopaldas and colleagues utilized the NIS database to compare the outcomes of CABG in patients with and without cirrhosis [56]. Adjusted analysis showed that presence of liver cirrhosis was independently associated with 7 fold higher odds of in-hospital mortality and 1.6 fold higher odds of complications. In a more recent study, Siraw studied 15,890 patients undergoing CABG and found that in-hospital mortality was significantly higher in patients with chronic liver disease (CLD), especially in those with cirrhosis (8.6% in cirrhotic CLD vs. 2.8% in non-CLD patients, *p* < 0.001) [57]. Cirrhotic patients showed elevated odds of cardiac complications such as cardiac arrest, cardiogenic shock, and ventricular fibrillation, as well as non-cardiac issues like acute kidney injury and gastrointestinal bleeding. Additionally, CLD patients had an extended hospital stay, with cirrhotic patients requiring an average of 11 days compared to 9 days in non-CLD patients​.

### Postoperative Complications

As with PCI, patients with cirrhosis undergoing CABG face significantly elevated risks of postoperative complications. Cirrhosis is often associated with coagulopathy, portal hypertension, and impaired immune function, all of which contribute to postoperative risks. Studies have consistently shown that these patients experience higher rates of complications such as infections, renal failure, respiratory failure, and bleeding. In addition to complications experienced following PCI, cirrhosis was also associated with increased risk of infections following cardiac surgeries [55, 58, 59]. Liu and colleagues investigated postoperative complications in cirrhotic patients undergoing cardiac surgery. The study defined complications as cardiovascular, pulmonary, gastrointestinal, renal, neurological diseases, or infections occurring after surgery. Analyzing data from seven studies involving approximately 1.5 million patients, they found that liver cirrhosis was associated with a 48% higher risk of postoperative complications (*p* < 0.01). Notably, cirrhotic patients faced more than double the risk of renal, gastrointestinal, and infectious complications following cardiac surgery [58]. 

### On-pump vs. Off-pump CABG (OPCAB)

The use of cardiopulmonary bypass in patients with liver cirrhosis remains contentious, with a consensus that it should be avoided in those with advanced disease due to its high-risk profile [60]. Cardiopulmonary bypass induces excess physiological, immunologic, and metabolic demands on the liver. It has been shown to stimulate the release of various vasoactive substances and cytotoxic compounds, which can disrupt coagulation, alter vascular resistance and permeability, and affect fluid balance and organ function. The catecholamines release during cardiopulmonary bypass reduce the hepatic perfusion, thus further compromising the liver function. The latter finding is supported by a study comparing on-pump against OPCAB, whereby there was a higher risk of hepatocellular injury, measured by liver transaminases, alkaline phosphatase, and total bilirubin levels, in the on-pump CABG group [61]. Additionally, factors such as hypothermia, hemodilution, and hypoperfusion during bypass may contribute to postoperative morbidity and mortality. Thus, avoiding the use of cardiopulmonary bypass may theoretically improve clinical outcomes by mitigating these adverse effects.

Supporting these findings, Gopaldas’s large-scale study of 3,046,709 CABG patients revealed that cirrhotic patients undergoing on-pump CABG had a significantly higher adjusted odds of mortality (aOR 6.9, 95% CI 2.8–17) and morbidity (aOR 1.6, 95% CI 1.3–2.0) compared to non-cirrhotic patients [56]. However, in the OPCAB group, cirrhosis was not associated with increased mortality or morbidity unless liver dysfunction was severe, in which case the odds of mortality rose (aOR 5.1, 95% CI 3.7–6.9). Consistent with these results, Hayashida’s study of cirrhotic patients undergoing cardiac surgery, with and without CPB, found that those with less severe cirrhosis (Child-Pugh class A) tolerated CPB without perioperative mortality, while those with more advanced disease (Child-Pugh classes B and C) faced substantial risks [62]. Together, these findings suggest that while CPB may be feasible in patients with milder liver dysfunction, its use in those with advanced cirrhosis substantially elevates perioperative risk, supporting a preference for OPCAB in this vulnerable population.

### Risk Prediction Models for Postoperative Mortality

Careful preoperative planning is critical for patient selection and optimization, given the significant risk of perioperative mortality in individuals with liver cirrhosis undergoing cardiac surgery. Both the Child-Pugh score and the Model for End-Stage Liver Disease (MELD) score have long been established as prognostic markers for hepatic decompensation and mortality in this population [63]. Emerging evidence suggests that these scoring systems not only predict long-term outcomes but also offer valuable insights into the risks of mortality following cardiac surgeries.

Traditionally, patients with liver cirrhosis are categorized into Child-Pugh classes A, B, and C. Studies have shown that as the Child-Pugh classification worsens, the postoperative mortality rate after cardiac surgery increases, with rates ranging from 0 to 11% for class A, 18-50% for class B, and 67-100% for class C [60, 64–66]. In a cohort study of 44 cirrhotic patients undergoing cardiac surgery with cardiopulmonary bypass, a Child-Pugh score of 8 or higher demonstrated 86% sensitivity and 92% specificity for predicting perioperative mortality [66]. Additionally, elevated MELD scores have been associated with increased length of hospital stay, higher in-hospital mortality, and a greater incidence of complications, including the need for renal replacement therapy and stroke [67, 68]. These findings highlight the importance of integrating liver function assessment into the perioperative risk stratification for patients with cirrhosis.

## PCI vs. CABG

The available data comparing revascularization strategies in patients with liver cirrhosis remain scarce. Marui and colleagues, utilizing a multicenter registry in Japan, investigated the outcomes of different revascularization approaches in this high-risk population. Their analysis demonstrated no significant difference in the risk of in-hospital mortality (*p* = 0.28) or all-cause mortality (*p* = 0.21) at follow-up between the revascularization strategies employed. Importantly, despite these findings, overall mortality rates were elevated, driven primarily by non-cardiovascular causes such as hepatic decompensation and malignancy. This observation raises the possibility that, in this population, complete revascularization may not meaningfully influence long-term outcomes, as the predominant drivers of mortality are non-cardiovascular causes. This emphasizes the importance of considering the broader clinical context in managing cirrhotic patients, where the balance of risks extends beyond cardiac events alone.

## Future Directions

The field of coronary interventions in patients with liver cirrhosis continues to expand, yet much remains to be explored in terms of long-term outcomes beyond the immediate risks of perioperative mortality and bleeding. Future research should place a greater emphasis on the chronic outcomes of coronary revascularization in this unique patient population, integrating the staging and severity of liver cirrhosis as critical prognostic factors. A shift toward precision medicine is imperative. Leveraging tools like the MELD score and detailed cirrhosis phenotyping may help refine individualized treatment strategies that could enhance patient outcomes. Large, prospective studies with longitudinal follow-up, rigorously stratified by cirrhosis severity, are essential to advancing this area. Moreover, the gap in evidence regarding secondary prevention post-revascularization calls for dedicated investigation to inform comprehensive care plans and improve the quality of life for these patients.

## Conclusion

In conclusion, managing coronary interventions in patients with liver cirrhosis presents unique challenges, with elevated risks of mortality, bleeding, and long-term complications. Current data emphasize the need for precision medicine approaches, incorporating liver disease severity and individualized treatment strategies to balance thrombotic and bleeding risks effectively. Despite some advancements, there remains a substantial gap in understanding long-term outcomes and optimal secondary prevention strategies for these patients.

## Key References

- Istanbuly S, Matetic A, Mohamed MO, Panaich S, Velagapudi P, Elgendy IY, et al. Comparison of Outcomes of Patients With Versus Without Chronic Liver Disease Undergoing Percutaneous Coronary Intervention. Am J Cardiol. 2021;156:32 − 8.

Findings from this study showed increased in-hospital mortality among patients with chronic liver disease undergoing PCI.

- Siraw BB, Patel P, Mehadi AY, Zaher EA, Tafesse YT. Association Between Chronic Liver Disease and Adverse In-Hospital Outcomes in Patients Undergoing CABG: A Propensity Score-Matched Analysis. Am J Cardiol. 2024;222:65–71.

Findings from this study highlight the increased in-hospital mortality and worse perioperative cardiac and non-cardiac complications among patients with chronic liver disease undergoing CABG.

## Data Availability

No datasets were generated or analysed during the current study.
